# Factors associated with the quality of life of family caregivers for leukemia patients in China

**DOI:** 10.1186/s12955-017-0628-6

**Published:** 2017-03-23

**Authors:** Hongjuan Yu, Limin Li, Chaojie Liu, Weidong Huang, Jin Zhou, Wenqi Fu, Yi Ma, Si Li, Yuying Chang, Guoxiang Liu, Qunhong Wu

**Affiliations:** 1 Department of Hematology, the First Affiliated Hospital of Harbin Medical University, Harbin, Heilongjiang 150001 China; 2 School of Psychology and Public Health, La Trobe University, Melbourne, VIC 3086 Australia; 3 School of Health Management, Harbin Medical University, 157 Baojian Road, Nangang District, Harbin, 150086 China; 4 Department of Hematology, the First Hospital of Harbin, Harbin, Heilongjiang 100730 China; 5 Department of Hematology, the Second Affiliated Hospital of Harbin Medical University, Harbin, Heilongjiang 150081 China

**Keywords:** Family caregiver, Leukemia, Quality of life, China

## Abstract

**Background:**

The leukemia affects not only the quality of life (QOL) of patients with the disease but also that of their family caregivers (FCs). The research studies on QOL of FCs for leukemia patients are limited. This study aimed to evaluate the QOL of FCs for leukemia patients in Heilongjiang province, China.

**Methods:**

A cross-sectional questionnaire survey was undertaken with 309 FCs for leukemia patients recruited from three hospitals in Heilongjiang province. The QOL of the participants was assessed using the Chinese version of WHOQOL-BREF. Multivariate regression models were established to determine the predictors of the QOL of FCs, including the socio-economic characteristics of patients and FCs, and the emotional distress, social support and family functions of FCs.

**Results:**

The FCs had low QOL scores in all four domains: 12.7 ± 2.8 for physical, 12.2 ± 2.5 for psychological, 13.2 ± 2.9 for social and 11.3 ± 2.5 for environment. Social support is a major predictor of the QOL of FCs, with a standardized β coefficient of “high support” ranging from 0.41 to 0.58 for the four domains, followed by family function (β = 0.37 ~ 0.44 for psychological, social and environmental domains). The FCs who were older, highly educated, had no religious belief, suffered from a higher level of emotional distress, and provided care to younger patients and the patients without insurance coverage had lower QOL than the others.

**Conclusion:**

The study provides some important insights into the QOL of FCs for leukemia patients. The QOL of FCs for leukemia patients is low and low levels of support to FCs are a major predictor of low QOL of FCs.

## Background

Cancer has a significant impact not only on the patients, but also on their family caregivers (FCs) [1]. The quality of life (QOL) of FCs for cancer patients is often lower than that of those caring for patients with other chronic illnesses [2]. Previous studies found that FCs of cancer patients often experience a high level of anxiety, depression, fatigue, hopelessness, fear, guilty, regret, sleep problems, and social isolation [3–11], some of which may not appear in those who care for patients without cancer.

FCs play a major role in how well a patient can manage his or her illness. They are often the primary source of social and emotional support for the patient [1]. The negative experience of caregivers can not only jeopardize their own QOL, but also compromise their ability to provide care [12]. The stress of FCs for patients with cancer leads to deteriorated physical health, immune function, and financial well-being, as well as psychological problems and sleep disturbances [12].

The QOL of FCs is determined by many factors, including those from the perspective of patients as well as those from the perspective of caregivers. Although some literature [12, 13] has reported an association between the QOL of FCs and the type of cancer of patients, most studies involved a sample with mixed types of cancer [14–17]. Such an approach has the advantage of increased sample size, but it may disguise potential differences in those who look after patients with different types of cancer. There is a paucity in the literature documenting cancer-specific QOL of FCs [18]. The few existing studies have focused on breast cancer, ovarian cancer, prostate cancer, and brain tumor [19–21].

This study aimed to identify factors associated with the QOL of FCs for leukemia patients in China. We chose leukemia as a focus for several reasons. First, leukemia patients have a high mortality. It is estimated that among the 352,000 people who develop leukemia every year globally, 265,000 (75.3%) die [22]. In China, about 75,300 new cases of leukemia were diagnosed and 53,400 leukemia patients died in 2015 [23]. Among the two most common types of leukemia in China, myeloid leukemia has significantly higher levels of incidence and mortality than lymphoid leukemia [24]. An advanced stage of cancer and symptom severity has been proved to be a reliable predictor of the QOL of FCs [4, 13]. A study in Japan demonstrated that the mothers caring for their children with leukemia have a lower QOL than the mothers caring for children without leukemia [25]. Second, the type of relationship between FCs and cancer patients has a significant influence on the commitment intensity of FCs [13]. Although leukemia occurs most often in older adults, it is also among the most common types of childhood cancer [26, 27]. This gives us a chance to examine the role of patient-FC relationships in the QOL of FCs. Previous studies [18, 25, 28] have restricted their samples to children with leukemia, including the one conducted in China [29], ignoring the fact that most leukemia patients are older adults. Although low QOL was found in FCs for children with leukemia, it is not clear whether there would be any difference for those who care for adult patients with leukemia. Third, there is a lack of study into the QOL of FCs for leukemia patients in China. A few studies undertaken outside of China have examined the QOL of FCs for patients with leukemia [18, 25, 28]. One study from Brazil with a small sample size reported low QOL of the mothers who provided care to their children with leukemia (*n* = 18) and non-Hodgkin lymphoma (*n* = 14) [18]. Another study conducted in Sri Lanka involving predominantly nuclear families showed low QOL of nearly 50% of FCs for leukemia patients in psychological, social and environmental domains [28].

In China, the traditional culture and Confucianism place a strong emphasis on the importance of family care [30–32]. It is a common practice in Chinese hospitals to require the family of a patient to arrange at least one person (either a family member or a paid care worker) to look after the patient. The duties include, but not limited to looking after personal hygiene of the patient, monitoring changes of patient conditions, keeping an eye on the progress of treatments such as *iv* drips, and providing foods. This can partly address the problem of nurse shortages, but it imposes great burdens on patient families. Limited financial resources may further exacerbate the heavy burden of family caregivers. Cancer is often seen as a tragedy, which is likely to attract extra attention and care from family members. We anticipated an even greater impact of childhood leukemia on the parents of patients due to the decades long “one child” policy which has been in place in China since the late 1970s.

## Methods

### Research setting

A cross-sectional survey was conducted in three hospitals in Harbin, the capital city of Heilongjiang province. The participating hospitals were selected because they are regional centers for the treatment of leukemia in Heilongjiang province. Heilongjiang, with a population of 38.33 million, ranked in the middle range of economic development among all provinces in China, with an average GDP of $6386 per capita in 2015 [33]. It was estimated that about 1663 patients suffer from leukemia every year in Heilongjiang [34].

### Data collection

Data were collected from July 2015 to February 2016. All of the leukemia patients admitted to the participating hospitals during the survey period and their primary family caregivers (FCs) were invited to participate in this study. Primary FCs were defined as a family member who takes the major responsibility of care for the patient and commits the largest proportion of time in the care for the patient without receiving any economic retribution.

A questionnaire was administered through face-to-face interviews in a private office in the hospitals. The interviewers were recruited from research students in Harbin Medical University and trained before embarking on the survey. The interviewers identified 349 eligible FCs for this study. Of these eligible FCs, 35 expressed no interest and declined to participate.

The participating FCs were encouraged to self-complete the questionnaire. But assistance from an interviewer was always available if necessary when, for example, the respondents had a low visual acuity or another disability. The interviewers reviewed the returned questionnaires and asked the respondents to complete missing items, if any. This resulted in an over 98% completion rate: of the 314 returned questionnaires, five contained missing data (in relation to age, religious belief, duration of caregiving, and education) and 309 questionnaires without missing data were included in final analyses.

### Measurements

The QOL of the FCs was the major concern of this study. The Chinese version of WHOQOL-BREF [35], which has been validated in various populations [15, 36, 37] was adopted to measure the QOL of the respondents. It contains 28 items, including 26 from the original version and two additional items specifically tailored to the Chinese context. The WHOQOL-BREF measures overall QOL and general health (2 items), physical health (7 items), psychological health (6 items), social relationships (3 items), and environment (8 items) using a five-point Likert scale. The item scores were added to calculate the domain scores, where a higher score indicates better QOL.

We hypothesized that the QOL of FCs is determined by the characteristics of the FCs and the patients, commitment intensity of care made by the FCs, emotional distress of the FCs, and support available to the FCs. This hypothesis was developed in line with several systematic reviews [12, 13].

We developed a questionnaire to collect data in relation to the characteristics of patients and their primary FCs. The characteristics of patients included age, sex, ethnicity, health insurance, time of diagnosis, and classification of leukemia. There are four major types of leukemia: acute lymphocytic leukemia (ALL), chronic lymphocytic leukemia (CLL), acute myelogenous leukemia (AML), and chronic myelogenous leukemia (CML). The characteristics of primary FCs included demographic characteristics (age, sex, ethnicity, marital status, employment, religion, and education), relationship to the patient, and household income.

Commitment intensity was measured by time spent per day (hours) of care while the patient stayed in the hospital and overall duration of care (months).

Emotional distress was assessed using the validated Hospital Anxiety and Depression Scale (HADS) [38], which is composed of two subscales measuring depression (7 items) and anxiety (7 items) in the prior week. Each subscale has a score ranging from 0 to 21, with a higher score indicating a more serious condition. The condition of depression or anxiety can be categorized as normal (0–7), mild (8–10), moderate (11–14), or severe (15–21) [39].

The level of support to the FCs was assessed by social support and family function. Social support was measured using the validated Social Support Rating Scale (SSRS) [40]. The SSRS captures subjective (4 items), objective (3 Items) and the use of social support (3 items). A summed score was calculated (ranging from 0 to 66), where a higher score indicates a higher level of social support. The level of social support was categorized as low (0–22), moderate (23–44), or high (45–66) according to the SSRS designers [41] for the purpose of statistical analyses.

Family function was measured using the validated family APGAR (adaptation, partnership, growth, affection, and resolve) scale [42, 43]. The APGAR scale asked respondents to rate their satisfaction with five statements (in relation to adaptation, partnership, growth, affection, and resolve, respectively) on a 3-point scale, ranging from 0 (hardly ever) to 2 (almost always). A summed score was calculated (ranging from 0 to 10), where a higher score indicates better family functioning. The family function can be categorized as severely dysfunctional (0–3), moderately dysfunctional (4–6), or highly functional (7–10) [39].

### Statistical analysis

The four domains of the WHOQOL-BREF were treated as dependent variables: physical, psychological, social relationship, and environmental.

Group comparisons on the QOL scores were made using student *t* tests (for two groups) or one-way ANOVA tests (for multiple groups). We tested the associations of the QOL (four domains) of the FCs with the characteristics of the patients (age, sex, ethnicity, type of leukemia, duration of illness, and medical insurance coverage), the characteristics of the FCs (age, sex, ethnicity, marital status, employment, relationship to patient, level of education, understanding of patient conditions, and religious belief), household income, commitment intensity of care (total amount of time and average hours per day committed to caregiving), emotional distress, and levels of support to the FCs (family function and social support).

The variables that showed a significant association (*p* < 0.05) with QOL were included in the multivariate linear regression models, with the four QOL domains serving as dependent variables. All independent variables entered into the regression models were coded or transformed into categorical measurements. We adopted an enter approach with a *p* value less than 0.05 being deemed as statistically significant.

Data entry and statistical analyses were conducted using the Statistical Package for Social Sciences (SPSS) 22 program for Windows.

## Results

The patients had a mean age of 35 years, with 24.6% being younger than 15 years. Slightly more than half (53.1%) of the patients were female; 95.5% were Han ethnicity; 90.6% were covered by medical insurance. AML was the most common diagnosis (52.8%), followed by ALL (31.1%), CML (13.6%) and CLL (2.6%). At the time of the survey, the patients had lived with leukemia (since diagnosis) for an average of 21 months (Table 1).

**Table 1:** Characteristics of patients and family caregivers (*n* = 309)

Characteristics	Caregivers	Patients
Gender (n, %)		
Male	140 (45.3%)	145 (46.9%)
Female	169 (54.7%)	164 (53.1%)
Age (years, mean±SD)	41.1±10. 8	35.4±20.8
Ethnicity		
Han	299 (96.8%)	295 (95.5%)
Other	10 (3.2%)	14 (4.5%)
Types of leukemia (n, %)		
ALL		96 (31.1%)
AML		163 (52.8%)
CLL		8 (2.6%)
CML		42 (13.6%)
Duration since diagnosis (Month, Mean±SD)		21.2±18.4
Duration of caregiving (Month, Mean±SD)	15.4±6.9	
Hours of caregiveing per day(Hour, Mean±SD)	17.8±7.2	
Understand of disease (n, %)		
Incompletely	7 (2.3%)	
Partial	192 (62.1%)	
Completely	110 (35.6%)	
Medical insurance (n, %)		
Yes		280 (90.6%)
No		29 (9.4%)
Annual household income (Yuan)		
≤40,000	168 (54.4%)	
40,001–79,999	131 (42.4%)	
≥80,000	10 (3.2%)	
Relationship to patient (n, %)		
Spouse	114 (36.9%)	
Parent	136 (44.0%)	
Child	43 (13.9%)	
Other	16 (5.2%)	
Level of education (n, %)		
No more than primary school	39 (12.6%)	
Middle or high school	204 (66.0%)	
University	66 (21.4%)	
Marital status (n, %)		
Married	291 (94.2%)	
Other	18 (5.8%)	
Employment (n, %)		
Employed	238 (77.0%)	
Retired	22 (7.1%)	
Unemployed	49 (15.9%)	
Religious belief (n, %)		
No	263 (85.1%)	
Yes	46 (14.9%)	
Anxiety (Mean±SD)	10.8±2.3	
Normal(n, %)	24(7.8%)	
Mild(n, %)	119(38.5%)	
Moderate (n, %)	149(48.2%)	
Severe(n, %)	17(5.5%)	
Depression (Mean±SD)	8.19±2.2	
Normal (n, %)	112(36.2%)	
Mild (n, %)	132(42.7%)	
Moderate (n, %)	65(21.0%)	
Severe (n, %)	0(0%)	
Social support (Mean±SD)	37.0±8.0	
Low(n, %)	10(3.2%)	
Moderate(n, %)	228(73.8%)	
High(n, %)	71(23.0%)	
Family function (APGAR score)	6.7±1.8	
Severely dysfunctional(n, %)	8(2.6%)	
Moderately dysfunctional(n, %)	134(43.4%)	
Highly functional(n, %)	167(54.0%)	

Most (54.7%) FCs were women. The majorities were parents (44.0%) or spouses (36.9%) of the patients, married (94.2%), employed (77.0%), and did not have religious belief (85.1%). The household income of the FCs was low compared with the local average, with more than half (54.4%) earning less than 40,000 Yuan a year (Table 1).

On average, the FCs had provided 15.4 months (SD = 6.9) of care for the patients. The duty of care was extremely intensive, with an average of 17.8 h (SD = 7.2) of commitments per day. Over the course, the FCs had developed a fairly good understanding about the disease. The majority of FCs admitted that they fully (35.6%) or partially (62.1%) understood the conditions of their patients.

The FCs experienced a high level of emotional distress, with 54% and 21% having a moderate/severe level of anxiety and depression, respectively. Slightly less than half of the families of the FCs were moderately (43.4%) or severely (2.6%) dysfunctional. The majority (73.8%) of FCs received a moderate level of social support (Table 1).

The FCs of leukemia patients had low QOL scores in all four domains: 12.7 ± 2.8 for physical, 12.2 ± 2.5 for psychological, 13.2 ± 2.9 for social and 11.3 ± 2.5 for environment. These scores were significantly lower compared with those found in the general populations of a national study [44] (*p* < 0.001) (Table 2).

**Table 2:** Quality of life scores of family caregivers (Mean ± SD)

Domain	Family Caregivers	General population	t	*P*
Physical	12.7±2.8	15.1±2.30	-15.09	0.000
Psychological	12.2±2.5	13.9±1.89	-11.70	0.000
Social	13.2±2.9	13.9±2.06	-4.60	0.000
Environment	11.3±2.5	12.1±2.08	-5.95	0.000

From the perspective of FCs, relationship to patient, depression, social support, and religious belief were found to be associated with all four domains of QOL in the univariate analyses. Those who were a parent or spouse, had no religious belief, suffered from depression, and received a lower level of social support had lower QOL. In addition, older age (>40) was associated with lower physical and social scores; lower education was associated with lower physical scores; and married FCs had a lower psychological score. Although length of caregiving was not linearly correlated with QOL scores, longer time spent on caregiving was associated with lower QOL in physical, psychological and environmental domains. Lower household income was associated with lower QOL in physical and environmental domains. A higher level of anxiety was associated with lower QOL in physical, psychological and social domains. Family dysfunction was associated with lower QOL in psychological, social and environmental domains (Table 3).

**Table 3:** Quality of life of family caregivers measured by WHOQOL-BREF

Characteristics	n	Physical	Psychological	Social	Environment
		Mean±SD	Mean±SD	Mean±SD	Mean±SD
Family caregivers					
Gender					
Male	140	12.8±2.9	12.2±2.7	13.3±3.1	11.5±2.6
Female	169	12.6±2.7	12.3±2.4	13.1±2.8	11.1±2.4
Age (years)					
≤40	156	**13.1±3.1**	12.4±2.8	**13.7±3.0**	11.3±2.6
>40	153	**12.2±2.4**	12.0±2.2	**12.6±2.7**	11.2±2.4
Relationship to patient					
Spouse	114	**12.5±2.5**	**11.8±2.2**	**12.5±2.6**	**11.1±2.2**
Parent	136	**12.1±2.6**	**11.8±2.4**	**13.2±2.8**	**11.1±2.6**
Child	43	**14.6±3.1**	**14.2±2.5**	**14.8±3.2**	**12.2±2.7**
Other	16	**14.0±3.4**	**12.8±3.4**	**13.3±3.9**	**11.7±3.6**
Level of education					
No more than primary school	39	**10.9±3.2**	11.4±2.4	12.2±3.4	10.5 ±2.5
Middle or high school	204	**12.9±2.7**	12.3±2.4	13.3±2.8	11.3±2.5
University	66	**13.1±2.6**	12.4±2.8	13.2±2.9	11.8±2.7
Ethnicity					
Han	299	12.7±2.8	12.2±2.5	13.2±2.9	11.3±2.6
Other	10	12.1±2.6	11.7±2.3	12.9±2.4	10.8±1.6
Religious belief					
No	263	**12.5±2.7**	**12.0±2.4**	**12.9±2.8**	**11.1±2.5**
Yes	46	**13.7±3.2**	**13.5±2.6**	**14.7±2.9**	**12.1±2.5**
Marital status					
Married	291	12.6±2.8	**12.1±2.5**	13.1±2.9	11.2±2.5
Other	18	13.6±2.9	**13.7±2.5**	13.7±2.5	12.1±2.1
Duration of caregiving (Months)					
≤6	112	12.5±3.0	**12.1±2.6**	13.2±3.2	11.3±2.7
6-12	69	12.8±2.8	**12.6±2.4**	13.4±2.8	11.5±2.2
13-24	70	12.6±3.1	**11.6±2.4**	12.6±2.9	10.8±2.5
>24	58	12.9±2.0	**12.7±2.6**	13.4±2.5	11.7±2.6
Time spent caregiving per day (Hours)					
0-12	127	**13.1±2.9**	**12.6±2.6**	13.4±3.2	**11.9±2.7**
13-24	182	**12.4±2.7**	**12.0±2.5**	13.0±2.7	**10.9±2.3**
Understanding of disease					
Lacking	7	13.1±1.1	12.6±2.7	13.5±2.5	13.1±1.7
Partial	192	12.6±2.8	12.2±2.3	13.0±2.9	11.2±2.4
Fully	110	12.8±3.0	12.3±2.8	13.4±2.9	11.2±2.8
Annual household income (Yuan)					
≤40,000	168	**12.2±2.9**	12.0±2.6	13.0±2.9	**10.9±2.4**
39,999-79,999	131	**13.1±2.7**	12.3±2.4	13.3±2.9	**11.7±2.6**
≥80,000	10	**14.0±2.7**	13.8±2.4	13.9±3.2	**12.5±2.7**
Employment					
Employed	238	12.8±2.8	12.3±2.6	13.3±3.0	11.4±2.6
Retired	22	12.7±1.5	11.9±1.8	11.9±1.2	11.4±2.0
Unemployed	49	12.3±3.2	12.1±2.5	13.0±2.9	10.9±2.5
Anxiety(HADS score)					
Normal	24	**14.4±2.9**	**14.2±2.7**	**15.1±3.0**	12.4±3.2
Mild	119	**13.0±2.7**	**12.6±2.4**	**13.7±2.8**	11.4±2.5
Moderate	149	**12.2±2.7**	**11.7±2.3**	**12.6±2.7**	11.1±2.4
Severe	17	**11.5±3.4**	**11.5±3.1**	**11.5±3.2**	10.6±2.4
Depression (HADS score)					
Normal	112	**13.2±2.3**	**12.7±2.4**	**13.7±2.8**	**12.6±2.5**
Mild	132	**12.7±2.8**	**12.1±2.4**	**13.1±2.8**	**10.9±2.2**
Moderate	65	**11.5±3.3**	**11.6±2.7**	**12.3±3.3**	**9.8±2.2**
Social support(SSRSG score)					
Low	10	**11.7±2.2**	**9.9±1.9**	**10.4±2.2**	**8.9±1.6**
Moderate	228	**12.1±2.6**	**11.7±2.3**	**12.6±2.8**	**10.8±2.2**
High	71	**14.8±2.4**	**14.3±2.2**	**15.3±2.2**	**13.2±2.4**
Family function(APGAR score)					
Severely dysfunctional	8	12.3±1.9	**10.7±1.5**	**11.2±2.8**	**9.8±2.3**
Moderate dysfunctional	134	12.3±2.9	**11.7±2.5**	**12.9±3.1**	**11.0±2.6**
Highly functional	167	13.0±2.8	**12.7±2.5**	**13.5±2.7**	**11.6±2.4**
Patients					
Gender					
Male	145	12.6±2.9	12.2±2.5	13.3±2.9	11.1±2.5
Female	164	12.8±2.8	12.2±2.6	13.1±3.0	11.4±2.5
Age (years)					
<15	75	**11.8±2.7**	**11.4±2.5**	13.4±2.6	**10.2±2.2**
≥15	234	**12.9±2.8**	**12.5±2.5**	13.1±3.0	**11.6±2.5**
Ethnicity					
Han	295	12.7±2.8	12.3±2.5	13.2±2.9	11.3±2.6
Other	14	12.0±2.4	11.1±2.4	12.7±2.3	10.9±1.3
Types of leukemia					
ALL	96	**12.2±2.7**	**11.4±2.6**	13.1±3.0	**10.6±2.7**
AML	163	**12.7±3.0**	**12.4±2.5**	13.1±2.9	**11.5±2.4**
CLL	8	**12.7±0.9**	**12.6±2.1**	13.3±1.6	**11.7±1.6**
CML	42	**13.7±2.6**	**13.2±2.2**	13.6±2.9	**11.9±2.4**
Duration since diagnosis (months)					
0-6	38	12.8±3.1	12.2±2.7	13.3±3.5	11.0±2.7
7-12	82	12.9±3.2	12.5±2.7	13.5±3.0	11.5±2.5
12-24	91	12.8±2.7	11.9±2.3	12.8±2.7	11.0±2.1
24	98	12.3±2.6	12.3±2.5	13.2±2.8	11.4±2.8
Medical insurance					
Yes	280	**12.8±2.9**	**12.3±2.6**	13.3±2.9	11.3±2.6
No	29	**11.3±2.1**	**10.9±1.8**	12.3±2.5	10.8±2.1

Bold indicates *P*<0.05 in group comparisons (Student’s t test for two groups or one-way ANOVA test for multiple groups)

From the patient perspective, a young patient age (<15) was associated with lower QOL of FCs in physical, psychological and environmental domains. ALL led to the lowest scores of FCs in physical, psychological and environmental domains among all types of leukemia. The FCs caring for patients without insurance coverage had lower scores in physical and psychological domains (Table 3).

The type of leukemia remained a predictor of QOL of FCs after controlling for other confounding factors in the multivariate models, but only for the psychological domain. CML and AML had a relatively less effect on the psychological health of FCs in comparison with ALL. Higher physical and environmental scores of FCs were found for those providing care for older patients. Patient insurance coverage showed a positive effect on the physical and psychological health of FCs (Table 4).

**Table 4:** Factors associated with quality of life of caregivers(*n* = 309)

	Physical			Psychological			Social			Environment		
	Adjusted β	t	*p*	Adjusted β	t	*p*	Adjusted β	t	*p*	Adjusted β	t	*p*
Family Caregivers												
Older age (vs. ≤ 40 years)	-0.18	-3.19	0.002				-0.06	-1.22	0.225			
Relationship to patient(vs.others)												
Spouse	-0.19	-1.80	0.073	-0.10	-1.02	0.310	-0.04	-0.35	0.724	-0.08	-0.84	0.403
Parent	-0.19	-1.70	0.090	-0.06	-0.63	0.531	0.03	0.28	0.781	0.09	0.82	0.416
Child	0.01	0.14	0.891	0.12	1.56	0.119	0.09	1.19	0.235	0.00	-0.05	0.963
Level of education(vs. ≤ primary school)												
Middle or high school	0.19	2.67	0.008									
University	0.15	1.91	0.057									
Religious belief(vs. no belief)	0.13	2.68	0.008	0.16	3.59	0.000	0.13	3.02	0.003	0.09	2.03	0.043
Married (vs.others)				0.07	1.64	0.101						
Duration of caregiving (months)				0.07	1.65	0.100						
13-24 hours spent caregiving per day (vs.0-12 hours)	0.03	0.63	0.528	0.03	0.64	0.522				-0.05	-0.99	0.323
Annual household income (vs. ≤40,000 Yuan)												
39,999-79,999	0.05	0.91	0.364							0.07	1.51	0.131
≥80,000	0.09	1.77	0.077							0.03	0.71	0.481
Anxiety (vs.normal)												
Mild	-0.19	-2.13	0.034	-0.16	-1.93	0.055	-0.12	-1.35	0.178			
Moderate	-0.26	-2.78	0.006	-0.27	-3.13	0.002	-0.22	-2.54	0.012			
Severe	-0.15	-2.44	0.015	-0.13	-2.33	0.021	-0.17	-2.97	0.003			
Depression(vs.normal)												
Mild	-0.15	-2.88	0.004	-0.04	-0.93	0.352	-0.04	-0.88	0.381	-0.22	-4.53	0.000
Moderate	-0.21	-3.97	0.000	0.00	-0.08	0.939	-0.05	-1.12	0.263	-0.14	-2.82	0.005
Family function (vs.highly functional)												
Severely dysfunctional				-0.37	-6.30	0.000	-0.44	-7.16	0.000	-0.42	-6.95	0.000
Moderate dysfunctional				-0.20	-3.63	0.000	-0.24	-4.09	0.000	-0.23	-3.95	0.000
Social support(vs. low)												
Moderate	0.12	1.12	0.262	0.28	2.81	0.005	0.35	3.53	0.000	0.26	2.60	0.010
High	0.41	3.74	0.000	0.52	5.17	0.000	0.58	5.60	0.000	0.51	4.96	0.000
Patients												
Older patients(vs.<15)	0.15	2.04	0.042	0.03	0.55	0.584				0.19	3.09	0.002
Types of leukemia(vs.ALL)												
AML	-0.02	-0.28	0.778	0.19	3.15	0.002				0.12	1.89	0.059
CLL	-0.02	-0.40	0.686	0.03	0.58	0.563				0.00	0.04	0.966
CML	0.07	1.18	0.239	0.14	2.53	0.012				0.03	0.56	0.573
Medical insurance(vs. no insurance)	0.11	2.32	0.021	0.09	2.21	0.028						
R^2^		0.40			0.50			0.46			0.46	
Adjusted R^2^		0.36			0.46			0.43			0.43	

Using a multivariate linear regression model (Enter) for significant (p<0.05) variables during univariate analyses

From the perspective of FCs, relationship to patients became insignificant in predicting the QOL of FCs in the multivariate models, although religious belief and social support remained a significant predictor of all four domains of QOL. In the regression models, social support was the biggest predictor of QOL of FCs: a high level of social support was associated with a 41–58% increase in the four domain scores compared with low support. This was followed by family function, where severe dysfunction was associated with a 37–42% decrease in the psychological, social and environmental scores of FCs. Depression was associated with lower physical and environmental scores, while anxiety was associated with lower scores of physical, psychological and social scores. Older age and higher educational attainments were also associated with lower physical scores. Marital status, household income, and duration and intensity of care commitment were insignificant for predicting QOL of FCs (Table 4).

## Discussion

### Increasing attention needs to be paid to the low QOL of FCs for leukemia patients

Lower QOL was found for the FCs of leukemia patients compared with the general populations in China [44]. This is consistent with the findings of other studies. A study in Japan showed that mothers caring for children with leukemia had lower scores across all eight domains of the Short Form 36, compared with mothers caring for children without leukemia [25]. Two studies of FCs for other cancer patients in China also revealed a lower QOL of FCs in comparison with the general adult populations [45, 46].

The low QOL of FCs deserves high attention, especially in a culture that highly values family input but has limited reliance on the social/professional care of patients [47, 48]. In China, family members play a major role in hospital care for patients. They undertake many duties that are otherwise delivered by nurses in western countries. This is evident in this study. We found that FCs spent an average of 17.8 h per day providing care for leukemia patients. The importance of family support has also attracted attention from researchers in Taiwan and Korea [49, 50].

It is widely accepted that the relationship between FCs and the patients they care for has a profound impact on the QOL of FCs. But this study failed to establish an association between the QOL of FCs and their relationship to patients. The FCs participating in this study are predominantly (more than 80%) parents or spouses of the patients, both having low but similar levels of QOL. However, the multivariate regression models do reveal a positive correlation between patient age and the QOL of FCs, indicating a worse QOL for those caring for young (<15 years) patients. This is different from the findings of another study of cancer patients, where younger patients were found to have a better QOL, which in turn, led to a better QOL of their caregivers [51]. Such a difference may be explained by the type of cancer. Leukemia is one of the most common cancers found in children. Childhood cancer is a devastating event for a Chinese family, especially when one family has only one child [52]. Most childhood leukemia are ALL [53] and we found that the FCs providing care for ALL had the worst QOL. This result is consistent with findings of other studies [54, 55]. In our study, 64% of the ALL patients were younger than 15 years and 98% of the child patients were cared by their parents. Although the therapeutic outcome of leukemia has improved considerably, the long duration and course of treatment, complications, and high medical bills continue to haunt many patients’ families [56, 57].

### Instrumental support can be essential for improving the QOL of FCs

The level of support to FCs plays a critical role in shaping the QOL of FCs for leukemia patients. Social support is associated with all four domains, while family function is a predictor of three domains. Family function and social support are the two biggest predictors of QOL of FCs, with a standard β coefficient ranging from 0.37 to 0.58. Previous studies reported social support as a significant predictor of the QOL of leukemia patients [58, 59]. But little attention has been paid to the role of social support in the QOL of FCs for cancer patients [60]. The social support obtained by the FCs in this study was already quite high, with a level comparable to the earthquake survivors who attracted national- and international-wide assistance [61]. However, it is concerning that the FCs for leukemia patients reported a high percentage (46%) of dysfunctional families.

Financial support may be able to help ease the burden of FCs. We found that patient insurance coverage is a predictor of higher QOL of FCs, in particular in relation to their physical and psychological functioning. In a study of FCs for children with leukemia in China, Tian and colleagues [29] also found a positive correlation between household income and the QOL of FCs. Medical treatments for leukemia are expensive, which can result in financial stress on patient families. Although many care duties are dumped to FCs or paid care workers as a result of shortages of nursing workforce, the social health insurance programs in China do not cover these expenses [62]. Families with financial difficulties not only face restrictions in their choice of treatment regimes, they are also less likely to hire paid workers to alleviate the burden of caregiving [50, 63].

### Psychological and emotional support may play a limited role for improving the QOL of FCs

Although emotional distress has been widely used for interpreting the low QOL of FCs for cancer patients [17, 64], this study demonstrates that it predicts some but not all of the QOL domains. Depression does not predict the psychological and social functioning of the FCs; whereas, anxiety does not predict the environmental domain of the FCs. Furthermore, the standard β coefficients of depression and anxiety are consistently small (0.14–0.27) in the regression models, indicating a weak association with QOL.

Although only a small proportion (14.9%) of FCs had a religious belief, those with a religious belief were found to have higher QOL. Two studies of FCs in Asian countries demonstrated a similar result [17, 50]. It was worth noting that religion has been spreading rapidly in China in recent years [65], albeit still at a relatively low level compared to other Asian countries, such as Korea (60.8%) [66]. Further studies are needed to tap into the role of psychological and emotional support on the QOL of FCs [60].

## Limitations

This study has several limitations. As a cross-sectional survey, it is not possible to make a causal inference about the associations between the investigated factors and the QOL of FCs. The participants were recruited from one province in China. Generalization of the findings should be cautious. However, the findings on the factors associated with the QOL of FCs are unlikely to be seriously influenced by the sample selection bias. This study did not explore the relationship between patient QOL and the QOL of FCs, simply because the QOL assessment instrument selected in this study cannot be applied to children.

## Conclusion

The study provides some important insights into the QOL of FCs for leukemia patients. The QOL of FCs for leukemia patients is low, which is determined by the characteristics of both patients and FCs. Low levels of support to FCs are a major predictor of low QOL of FCs. Particular attention should be paid to those who provide care to child patients and patients without insurance coverage, and those who experience family dysfunctions.

## Abbreviations

ALL: Acute lymphocytic leukemia
AML: Acute myelogenous leukemia
CLL: Chronic lymphocytic leukemia
CML: Chronic myelogenous leukemia
FCs: Family caregivers
HADS: Hospital Anxiety and Depression Scale
QOL: Quality of life
SPSS: Statistical Package for Social Sciences
SSRS: Social Support Rating Scale
